# The Ponseti Method vs. Surgical Treatment for Idiopathic Clubfoot: A Prospective Long-Term Follow-Up

**DOI:** 10.3390/children11121422

**Published:** 2024-11-26

**Authors:** Martin Svehlik, Bernhard Guggenberger, Michael Stöckler, Sebastian Klim, Tanja Kraus

**Affiliations:** 1 Pediatric and Adolescent Unit, Department of Trauma and Orthopedics, Medical University of Graz, 8036 Graz, Austria; martin.svehlik@medunigraz.at (M.S.); tanja.kraus@medunigraz.at (T.K.); 2 Institute of Human Movement Science, Sport and Health, University of Graz, 8010 Graz, Austria; 3 Institute of Physiotherapy, Joanneum University of Applied Sciences, 8020 Graz, Austria; 4 Department of Trauma and Orthopedics, Medical University of Graz, 8036 Graz, Austria

**Keywords:** clubfoot, Ponseti method, surgical treatment, conservative treatment, rehabilitation, pediatric orthopedics

## Abstract

**Background:** Idiopathic clubfoot is a common skeletal deformity in newborns that can cause functional limitations, pain, and reduced quality of life. The Ponseti method has become the gold standard for clubfoot treatment, replacing previous extensive surgical approaches. However, there is a lack of prospective long-term data comparing surgical and Ponseti treatments. **Methods:** This is a follow-up on a single-center, prospective, randomized clinical trial that started in 2001. The aim of this study was to compare the Ponseti method with surgical treatment. The current report presents the long-term results at adulthood of 12 clubfeet (9 subjects) in the Ponseti group and 9 clubfeet (5 subjects) in the surgical group. The evaluation included morphology, function, and quality of life, which were assessed through gait analysis, X-rays, and standardized questionnaires (FRS, ICFSG, AOFAS, SF-36). A Functional Rating System for clubfeet was defined as the primary outcome. **Results:** The Ponseti group had higher Functional Rating System scores, better ankle dorsiflexion mobility, and lower pain levels. Additionally, they reported better quality of life according to the Short Form 36 survey. However, neither group achieved radiographically normal foot morphology. **Conclusions:** The 18-year follow-up results indicate that the Ponseti method produces superior outcomes in terms of mobility, gait, function, and quality of life when compared to surgically treated feet, despite the presence of persistent morphological deviations.

## 1. Introduction

Congenital clubfoot, also known as talipes equinovarus, is one of the most common foot deformities in newborns, occurring in approximately 1 in 1000 live births [1]. This complex deformity includes equinus, hindfoot varus, forefoot adductus, and supination of the foot [2,3]. If left untreated, clubfeet can lead to progressive deformity and discomfort [4]. Untreated clubfeet can adversely impact educational and employment opportunities, as well as self-esteem and personal development [5,6]. The primary goal of clubfoot treatment is to attain a plantigrade, functionally sound foot with a normal range of motion (ROM).

Historically, the treatment of clubfeet has been predominantly surgical, involving extensive surgical release after prolonged casting in infancy [7]. However, long-term studies have shown limitations in range of motion, foot function, and quality of life in surgically treated patients [8]. In addition, patients who undergo fewer surgical procedures tend to have better outcomes, highlighting the potential of conservative treatment approaches. In this context, the Ponseti method, a conservative treatment, has emerged as the global gold standard for clubfoot correction [9]. Known for its simplicity, cost-effectiveness and high success rate, the Ponseti method involves a combination of manipulative techniques, casting, and bracing [3].

Although numerous studies have shown the favorable outcomes of the Ponseti method [10], there are limited data documenting its long-term advantages in terms of morphology, function, and, quality of life compared to the formerly used surgical approach in long-term trials. The aim of this prospective study was to evaluate the long-term outcomes of clubfoot treatment 18 years post-intervention in young adults by comparing the Ponseti method with surgical treatment.

## 2. Materials and Methods

### 2.1. Study Setting and Design

This is a single-center, randomized, controlled, non-blinded, prospective, long-term, intention-to-treat study designed to evaluate the long-term outcomes of clubfoot interventions. The primary outcome measure is the Functional Rating System (FRS), which was chosen to assess treatment efficacy [11]. This paper presents the results of the final long-term follow-up at the beginning of adulthood of patients with clubfoot who underwent treatment using either the Ponseti method or surgical intervention [12,13]. The presented follow-up was planned for 18 years after treatment onset. Ethical approval for this final follow-up was granted by the local institutional review board (IRB00002556, EK Nr. 24-221).

### 2.2. Participants

The initial trial was planned with a sample size of 23 individuals per group, based on the anticipated outcomes for the FRS, with a power of 0.9 and an alpha error of 0.05. However, recruitment was halted by the local ethical committee following an interim report. This decision arose due to the greater need for invasive surgeries in the surgical group to correct the clubfoot, in contrast to simple tenotomies in the Ponseti group. In the initial study, 12 clubfeet in 9 individuals were enrolled in the Ponseti group, while 16 clubfeet in 10 individuals were included in the surgical group. Random allocation to the groups was performed using a randomization table, and bilateral clubfeet received the same treatment. For more information on the recruitment procedure, refer to [12].

**Figure 1:**
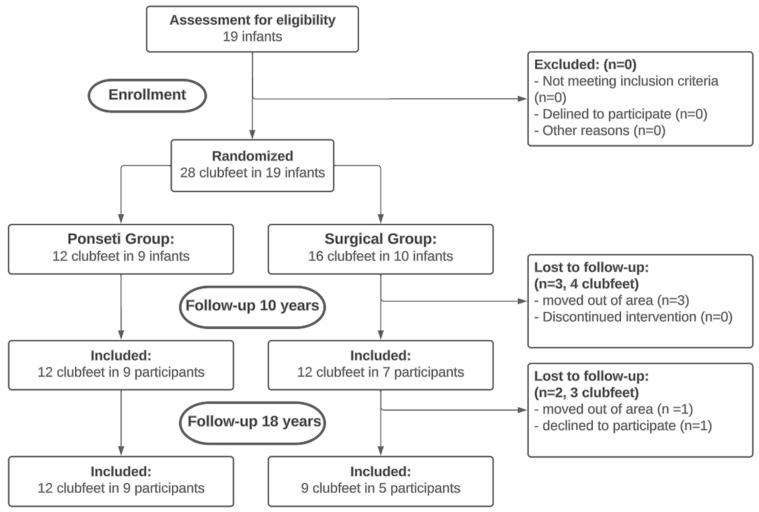
Consort flow diagram. Provides an overview of group allocations and losses to follow-up.

All participants, from the 10-year follow-up study, were invited to participate in the presented study. In total, 12 clubfeet in 9 individuals were included in the Ponseti group and 9 clubfeet in 5 individuals in the surgical group (Figure 1). The average follow-up time was 18.0 years. Written informed consent was obtained from all participants aged 18 years or older. For minors (<18 years), parental consent was also obtained. Exclusion criteria included refusal to participate in the trial.

### 2.3. Demographics and Description of Study Population

Severity of the clubfeet was assessed using the Pirani score at the onset of the treatment [14,15]. No differences in Pirani score at treatment onset and in demographic characteristics at the start of the third follow-up were observed between groups (Table 1).

In the Ponseti group, four clubfeet required additional treatment due to forefoot supination during the swing phase (Table 1). Two clubfeet underwent a second achillotomy, while two clubfeet underwent a tibialis anterior transfer. In contrast, the surgical group had six clubfeet requiring additional surgeries. Four clubfeet underwent an anterior tibialis anterior transfer, and two clubfeet required a major revision surgery involving partial release. Additionally, two clubfeet underwent a cuboid and cuneiform osteotomy following the tibialis anterior transfer.

During the follow-up period, two clubfeet from two participants in the Ponseti group deviated from the planned treatment protocol, resulting in observed deformity relapse. This prompted parental deviation from the intended conservative approach. However, following the intention-to-treat principle, these cases remained assigned to the Ponseti group.

### 2.4. Treatment Regime

Participants in the Ponseti group underwent weekly treatments consisting of manipulative techniques and application of an above-knee cast, following the Ponseti method [3]. After Achilles tenotomy, a final cast was applied for three weeks, followed by orthotic management once the desired correction was achieved. All patients were provided with abduction braces set at 70 degrees of external rotation for the affected foot, with unilateral cases adjusted to 45 degrees for the healthy foot. Orthotic treatment was discontinued at the age of two, following the Ponseti protocol. Afterwards, custom-molded shoes were used for daily wear, in accordance with the established house protocol at that time. Participants in the surgical group received casting treatment following Johann Bösch’s method [16], which continued until the age of 6–8 months or until a minimum foot length of 8 cm was attained. The remaining foot deformities were treated using the Cincinnati approach [17] through posteromedial release, followed by casting. The hospital stay averaged six days, and there were no reported neurovascular or skin complications. The plaster casts were removed six weeks after the surgery, and rigid ankle foot orthoses were used for night-time splinting until the age of 36 months. Additional information regarding treatment protocols can be found in a previous publication outlining this prospective study [12].

### 2.5. Outcome Measures

During the current follow-up, all participants underwent thorough clinical examinations. Passive ankle joint ROM was measured using a goniometer with the knee joint both extended and flexed. Furthermore, foot morphology was evaluated through standing foot X-rays. The primary outcome measure was the FRS, which was specifically developed for evaluating clubfoot treatment. The assessment includes several subscales that evaluate satisfaction, function, pain, heel position during stance, passive motion, and gait. Higher scores indicate better outcomes, and a total score is generated by combining all subscale results. Scores between 90 and 100 points are classified as excellent, 80 and 89 as good, 70 and 79 as fair, and below 70 as poor [11].

To evaluate function and pain, the International Clubfoot Study Group (ICFSG) scale [18] and the American Orthopaedic Foot and Ankle Society (AOFAS), Ankle-Hindfoot Scale, and Ankle-Midfoot Scale [19,20] were used. The Short Form 36 General Health (SF-36) questionnaire was administered to assess psychosocial quality of life [21,22].

All participants underwent clinical gait analysis using a 3D motion capture system (Vicon Motion Systems, Oxford, UK) and the modified Cleveland markerset [23]. The kinematic data were used to calculate the minimum, maximum, and mean values of relevant parameters during the stance phase. Additionally, the overall ROM was computed over the gait cycle. Joint moments and power were determined to provide a comprehensive understanding of gait mechanics. To compare overall gait pattern and performance, the Gait Profile Score (GPS) was calculated for each participant [24].

### 2.6. Statistical Analysis

Upon data collection, statistical analysis was performed utilizing Statistica (14.1.0, Statsoft, Tulsa, OK, USA). Descriptive statistics were used to provide an initial overview of the data. The Mann–Whitney-U test was employed to compare outcomes among the groups, while the Chi-squared test was utilized for categorical variables. The alpha level was set at 0.05. Data are expressed as mean ± standard deviation for both the Ponseti and surgical cohorts, accompanied by the corresponding *p*-value derived from intergroup comparisons.

## 3. Results

### 3.1. Standardized Scoring Systems

The Ponseti group showed better results in the FRS total score, as well as in its individual subscales of satisfaction, function, pain, and passive motion as depicted in Table 2.

When comparing the groups based on their ICFSG score, it was found that the surgical group had inferior results compared to the Ponseti group in both total score and subscales of morphology and functional evaluation (Table 3). However, there was no significant difference in the radiologic evaluation between the two groups. The Ponseti group had better outcomes overall, with superior function, lower pain levels, and higher scores on both the AOFAS Ankle-Hindfoot and AOFAS Midfoot Scales (Table 4). There was no significant difference in the alignment subscale of either AOFAS scale between the two groups.

The Ponseti group scored better in the subscales of general health, health change, and pain of the SF-36 questionnaire. Although the Ponseti group demonstrated a trend towards higher values in the physical functioning domain, it did not reach statistical significance. Furthermore, no differences were found between the groups for the subscales of role limitations, social functioning, emotional wellbeing, and energy/fatigue (Table 5).

### 3.2. Clinical Examination

In the clinical examination, the Ponseti group exhibited superior dorsiflexion with both extended and flexed knee positions. However, there was no significant difference between groups observed in plantar flexion in those two positions (Table 6).

### 3.3. Radiological Measurements

Regarding radiological measurements, the Ponseti group exhibited a higher lateral talo-calcaneal angle when compared to the surgical group. Additionally, flat-top talus deformity was more frequently observed in the surgical group. However, no significant differences were found for the antero-posterior talo-calcaneal angle and calcaneal pitch angle (Table 7).

### 3.4. Gait Analysis

The Ponseti group demonstrated a superior functional ankle range of motion throughout gait. Additionally, the Ponseti group exhibited higher ankle moments and generated more power in the sagittal plane during toe-off (Table 8). In terms of knee and hip kinematics and kinetics, there were no significant differences between the groups. No significant differences were observed in the GPS between the two groups (5.84 ± 1.30 for the Ponseti group vs. 7.38 ± 1.38 for the surgical group, *p* > 0.052).

## 4. Discussion

The aim of this investigation was to evaluate the long-term effects of the Ponseti method for correcting clubfoot on functionality, anatomical structure, and quality of life in comparison to surgical interventions in adult individuals. The data indicate that the Ponseti method results in better outcomes in terms of mobility, walking capabilities, and overall quality of life. Deviations from normal radiographic foot morphology were observed in participants from both treatment cohorts when measured against standard physiological benchmarks.

The Ponseti method is widely regarded as the most effective treatment for clubfoot in recent years. Numerous studies have confirmed its efficacy, particularly in terms of short-term outcomes [25]. Furthermore, retrospective analyses of long-term efficacy have supported these findings [10]. However, there is a lack of prospective studies comparing the long-term outcomes of the Ponseti method with surgical interventions in young adults. Prospective studies are crucial for ensuring that therapeutic results for clubfoot treatment remain constant throughout skeletal maturation and can withstand the increasing biomechanical forces exerted on the feet during the transition to ambulation. Ponseti emphasized the importance of such studies and showed that individuals with clubfeet can achieve satisfactory function and mobility even into adolescence and young adulthood [3].

The Ponseti group had superior outcomes regarding ROM, particularly ankle dorsiflexion. This finding is consistent with previous research on conservative clubfoot management in adolescents [26,27]. Notably, similar to retrospective long-term follow-up, our study showed persistent structural deviations in both treatment groups [10], despite improved ROM in the Ponseti group. Invasive surgical procedures can generate scar tissue that may hinder ankle mobility and restrict range of motion during growth [28]. It is possible that scar tissue plays a significant role in reduced mobility among clubfoot patients who have undergone surgery due to the early initiation of treatment.

Although, the radiographic evaluations showed minimal distinctions between the groups, there was a noticeable prevalence of flat-top talus in the surgical cohort. The reduced range of motion may be explained by the documented evidence that a flatter talus adversely affects ankle joint mobility [29,30]. Additionally, the persistent deformities evident in the radiological assessments of both treatment groups suggest that inferior mobility among patients who underwent surgical intervention could also be attributed to the formation of scar tissue subsequent to surgery.

For individuals with clubfeet, activities of daily living and ambulation are of paramount importance, going beyond the isolated metrics of range of motion and radiological morphology. By employing the function subscales of the FRS, the AOFAS Hindfoot and Midfoot Scales, as well as the ICFSG evaluation, we were able to comprehensively assess and delineate the superior functional outcomes associated with Ponseti-treated clubfeet. Although the FRS did not detect any differences in gait, objective gait analysis revealed significant differences in ankle kinematics and kinetics between the two groups. The Ponseti group demonstrated increased ankle range of motion during walking, consistent with the favorable findings from clinical range of motion assessments. The surgical cohort showed diminished ankle moments and powers, which signify a substantial functional limitation and impede normal propulsion. The reduced ankle power during push-off in the surgical cohort may be due to inadequate lever arm mechanics caused by greater instability in overcorrected clubfeet. These findings align with prior research that elucidates the impact of clubfoot treatment on gait dynamics [26,31]. GPS did not show any difference between both groups. This is not surprising, as the GPS might not represent the changes in the ankle kinematics adequately [32]. While clubfoot treatment has a noticeable effect on ankle joint mechanics, its impact on knee and hip joints is relatively minor.

Beside gait analysis, this study employed several patient-related outcome measures, with assessing perceived pain levels being a crucial component. Individuals within the surgical cohort consistently reported significantly higher levels of pain across all scales used. These findings are consistent with a previous study that compared pain levels between Ponseti clubfoot treatment and surgical intervention [31]. The increased pain experienced after surgical treatment may be due to limited range of motion in the ankle and subtalar joints, resulting in mechanical overload and subsequent discomfort.

Mobility, functional capacity, and pain perception are important factors in assessing quality of life. Limitations faced by those who underwent surgical treatment may significantly impede daily functioning. Individuals who underwent surgical intervention reported lower levels of satisfaction and quality of life, consistent with diminished functional outcomes, gait parameters, and pain levels. Additionally, the Ponseti group reported better outcomes in satisfaction scores, as evidenced by the FRS subscale, and the group demonstrated superior scores in the SF-36 assessment, particularly in domains concerning general health and health-related changes. Prior research has reinforced these findings, highlighting the superior quality of life associated with Ponseti treatment in comparison to surgical interventions [26,31].

In addition to previously mentioned findings, it is important to address the supplementary interventions observed in both treatment cohorts. In the Ponseti group, four clubfeet required further intervention due to forefoot supination during the swing phase. In contrast, the surgical cohort required additional surgery, with six clubfeet undergoing such procedures. Although some subjects in the Ponseti group may require subsequent surgical interventions, the Ponseti method still remains less invasive than surgical treatments.

There are several limitations that need to be considered carefully when interpreting the results presented in this paper. The most significant of these is the modest sample size. Following an interim report, the ethical committee halted participant recruitment due to the larger and more invasive interventions required in the surgical group compared to the Ponseti group. The decision was made after enrolling only half of the initially planned subjects, which prevented the attainment of the calculated sample size and a power of 0.9, as outlined by Zwick et al. [13]. The trial was also affected by dropout, with two participants from the surgical arm leaving the trial, reducing the sample size for long-term follow-up. Furthermore, the lack of blinding among assessors to the treatment allocation presents a limitation. Blinding was impractical due to the conspicuous scarring evident in the surgical group. Therefore, the findings of this study should be interpreted cautiously, recognizing their limited generalizability to the broader population. However, it is important to note that this study is the only prospective randomized trial with such a long follow-up duration in the existing literature.

## 5. Conclusions

While neither of the two compared treatment modalities resulted in the attainment of physiologically normalized radiological parameters, the Ponseti method emerged as the preferred approach due to its superior outcomes in terms of mobility, pain alleviation, functional capacity, and overall quality of life. Thus, the Ponseti method is an effective and minimally invasive treatment modality for patients with idiopathic clubfoot.

## Figures and Tables

**Table 1:** Description of group demographics (Major revision surgery = posteromedial release in 2 participants of the Ponseti group (opted out of assigned treatment) and revision surgery in the surgery group. C/O wedge OT = Opening wedge medial cuneiform osteotomy and closing wedge cuboid osteotomy).

	Ponseti Group	Surgical Group
Sample size (Clubfeet)	12	9
Unilateral: Bilateral	6:3	3:2
Sex (female:male)	5:7	1:4
Age at the Follow-up in years (mean ± SD)	18.1 ± 0.29	18.0 ± 0.76
Pirani Score at birth (mean ± SD)	3.5 ± 1.89	3.9 ± 0.88
Tibialis anterior transfer (N)	2	4
Additional achillotomy	2	0

**Table 2:** Result of the Functional Rating System (SD = standard deviation). Significant differences between groups are highlighted bold.

Functional Rating System					
	Ponseti Group		Surgical Group		
	Mean (±SD)	Median	Mean (±SD)	Median	*p*-Value
Satisfaction	20.00 (±0.00)	20	16.00 (±4.00)	16	***p* = 0.012**
Function	20.00 (±0.00)	20	16.00 (±3.46)	16	***p* = 0.012**
Pain	28.50 (±2.71)	30	18.67 (±7.62)	18	***p* = 0.003**
Position Heel standing	9.17 (±1.95)	10	8.89 (±2.20)	10	*p* = 0.859
Passive Motion	5.92 (±2.11)	7	3.00 (±2.40)	2	***p* = 0.012**
Gait	9.67 (±1.15)	10	9.11 (±1.76)	10	*p* = 0.619
Total	93.25 (±3.79)	93.5	71.67 (±12.86)	73	***p* < 0.001**

**Table 3:** Results of the ICFSG scale (SD = standard deviation). Significant differences between groups are highlighted bold.

International Clubfoot Study Group Scale					
	Ponseti Group		Surgical Group		
	Mean (±SD)	Median	Mean (±SD)	Median	*p*-Value
Morphology	2.33 (±1.15)	2.00	3.89 (±1.36)	4.00	***p* = 0.014**
Functional Evaluation	0.83 (±1.34)	0.00	3.67 (±2.06)	3.00	***p* = 0.004**
Radiologic Evaluation	5.00 (±1.81)	5.00	6.67 (±2.35)	5.00	*p* = 0.145
Total	8.17 (±2.21)	7.50	14.22 (±3.67)	12.00	***p* = 0.001**

**Table 4:** Results of the AOFAS Ankle-Hindfoot and Midfoot Scale (SD = standard deviation). Significant differences between groups are highlighted bold.

American Orthopaedic Foot and Ankle Society					
**Ankle-Hindfoot Scale**					
	**Ponseti Group**		**Surgical Group**		
	**Mean (±SD)**	**Median**	**Mean (±SD)**	**Median**	***p*** **-Value**
Pain	37.50 (±4.52)	40.00	25.56 (±11.30)	30.00	***p* = 0.0062**
Function	49.50 (±1.24)	50.00	43.00 (±5.70)	43.00	***p* = 0.0062**
Alignment	7.50 (±2.61)	5.00	5.00 (±0.00)	5.00	*p* = 0.0597
Total	94.50 (±6.79)	97.50	73.56 (±12.46)	76.00	***p* = 0.0003**
**Midfoot Scale**					
	**Ponseti Group**		**Surgical Group**		
	**Mean (±SD)**	**Median**	**Mean (±SD)**	**Median**	***p*** **-value**
Pain	37.50 (±4.52)	40.00	25.56 (±11.30)	30.00	***p* = 0.0062**
Function	43.42 (±2.19)	45.00	39.11 (±5.62)	40.00	***p* = 0.039**
Alignment	11.50 (±3.66)	8.00	8.00 (±0.00)	8.00	*p* = 0.0597
Total	92.42 (±8.44)	94.00	72.67 (±14.16)	77.00	***p* = 0.0006**

**Table 5:** Results of the Short Form 36 questionnaire (SD = standard deviation). Significant differences between groups are highlighted bold.

Short Form 36 Questionnaire					
	Ponseti Group		Surgical Group		
	Mean (±SD)	Median	Mean (±SD)	Median	*p*-Value
General Health	91.67 (±12.12)	100	77.78 (±17.34)	85	***p* = 0.036**
Health Change	60.42 (±16.71)	50	38.89 (±13.18)	50	***p* = 0.017**
Pain	99.17 (±2.89)	100	78.33 (±31.99)	90	***p* = 0.023**
Physical Functioning	97.5 (±7.23)	100	92.22 (±10.03)	95	*p* = 0.070
Role limitations	100.00 (±0.00)	100	88.89 (±22.05)	100	*p* = 0.41
Social functioning	92.42 (±8.44)	100	86.11 (±17.05)	100	*p* = 0.46
Emotional wellbeing	75.33 (±21.43)	80	81.78 (±10.02)	80	*p* = 0.67
Energy/fatique	59.58 (±24.54)	70	59.44 (±19.91)	55	*p* = 1.000

**Table 6:** Results of mobility measurements (DF = dorsiflexion, PF = plantarflexion, SD = standard deviation). Values of the mobility measurement are presented in degrees. Significant differences between groups are highlighted bold.

Mobility Measurements					
	Ponseti Group		Surgical Group		
	Mean (±SD)	Median	Mean (±SD)	Median	*p*-Value
DF Extended Knee	9.58 (±4.74)	10.00	4.44 (±4.48)	3.00	***p* = 0.043**
PF Extended Knee	50.25 (±15.31)	50.00	50.11 (±16.21)	50.00	*p* = 0.97
DF Flexed Knee	13.92 (±5.14)	15.00	10.11 (±11.88)	10.00	***p* = 0.043**
PF Flexed Knee	54.75 (±16.85)	52.00	48.33 (±24.59)	50.00	*p* = 0.59

**Table 7:** Results of radiological measurements (SD = standard deviation). For flat-top talus, a value of 1 represents yes and a 0 no. Significant differences between groups are highlighted bold.

Radiological Measurements					
	Ponseti Group		Surgical Group		
	Mean (±SD)	Median	Mean (±SD)	Median	*p*-Value
Talo-calcaneal angle (degrees)	19.50 (±13.67)	22.00	14.89 (±8.49)	13.00	*p* = 0.189
Talo-calcaneal angle (degrees)	39.58 (±5.88)	40.00	21.78 (±11.51)	26.00	***p* = 0.0007**
Calcaneal Pitch (degrees)	19.17 (±5.81)	18.50	13.11 (±6.99)	13.00	*p* = 0.051
Flat-Top Talus	0.25 (±0.45)	0.00	0.89 (±0.33)	1.00	***p* = 0.016**

**Table 8:** Overview of kinematic and kinetic gait analysis parameters. (HF = Hindfoot, FF = Forefoot, ROM = Range of motion, GC = Gait Cycle, SD = standard deviation). Significant differences between groups are highlighted bold.

Gait Analysis					
	Ponseti Group		Surgical Group		
	Mean (±SD)	Median	Mean (±SD)	Median	*p*-Value
Sagittal					
Ankle ROM over GC (degrees)	32.72 (±7.06)	33.38	22.45 (±3.9)	22.49	***p* = 0.004**
Ankle moment along GC (Nm/kg)	1.39 (±0.08)	1.40	1.18 (±0.21)	1.26	***p* = 0.019**
Ankle power along GC (W/kg)	3.02 (±0.71)	2.91	1.70 (±0.56)	1.49	***p* = 0.002**
Transversal					
Mean foot rotation in stance (degrees)	8.82 (±7.97)	11.45	1.42 (±19.36)	7.93	*p* = 0.451

## Data Availability

The data presented in this study are available on request from the corresponding author due to legal reasons.
